# Multiple stressors and stressor identification across assessment methods: findings from a psychosomatic rehabilitation sample within the ICD-11 adjustment disorder framework

**DOI:** 10.1080/21642850.2026.2714623

**Published:** 2026-08-06

**Authors:** Alexa Alica Kupferschmitt, Volker Köllner, Rahel Bachem

**Affiliations:** a Psychosomatic Rehabilitation Research Group, Department of Psychosomatic Medicine, Center for Internal Medicine and Dermatology, Charité – Universitätsmedizin Berlin, Berlin, Germany; b Department of Psychosomatic Medicine, Seehof Rehabilitation Center, Teltow, Germany; c Competence Center for Mental Health – Eastern Switzerland University of Applied Sciences, St. Gallen, Switzerland

**Keywords:** Adjustment disorder, ICD-11, trigger events, stressor identification, diagnostic validity

## Abstract

**Background:**

Adjustment disorder (AjD) is defined in ICD-11 by core symptoms of preoccupation and failure to adapt in response to a clearly identifiable stressor. Disorder-specific instruments, including the self-report questionnaire Adjustment Disorder - New Module 20 (ADNM-20) and the Composite International Diagnostic Interview - Module Adjustment Disorder (CIDI-AD), have been developed to operationalize these core features. However, it remains unclear to how consistently triggering events are identified across different assessment approaches. The present study aimed to identify the most frequent triggering events associated with AjD symptoms in psychosomatic rehabilitation and to examine the concordance of stressor identification across assessment approaches.

**Method:**

The ADNM-20 was administered to a clinical sample of psychosomatic inpatients (N = 2063). The CIDI-AD interview was conducted with a random subsample (*n* = 373) on the same day or the following day. Stressor categories (work, own illness, family, illness/death in family, other) were coded to enable comparison of stressor categories across instruments.

**Results:**

Work-related problems were the most common triggers in both instruments, followed by own illness. Approximately half of the participants (≈ 49%) reported different primary stressors in the questionnaire and the interview. On average, participants reported nine stressors in the ADNM-20 (range 1–19 events) and five stressors in the CIDI-AD (range 1–19 events), indicating that many individuals in psychosomatic rehabilitation experience multiple concurrent stressors.

**Conclusion:**

The findings indicate that stressor identification varies substantially across assessment methods in psychosomatic rehabilitation settings. The frequent occurrence of multiple, overlapping stressors suggests that AjD-related symptoms may not always be attributable to a single dominant event. These results underscore the importance of considering cumulative stress exposure in AjD assessment and highlight the need for future research to clarify how the ICD-11 single-stressor assumption applies in populations with concurrent stressors.

## Article highlights

- Participants in psychosomatic rehabilitation frequently reported multiple concurrent stressors across life domains.
- Agreement regarding the primary stressor differed substantially between questionnaire- and interview-based assessment.
- Findings suggest that identifying a single ‘main’ stressor may be challenging in clinically complex contexts.
- Results highlight the importance of considering cumulative stress exposure in ICD-11 AjD assessment.

Adjustment disorder (AjD) is a maladaptive psychological response to identifiable psychosocial stressors, characterised by distress that exceeds what would normally be expected given the nature of the stressor. In contrast to post-traumatic stress disorder (PTSD), which follows traumatic events of an existential or life-threatening nature, AjD typically develops in response to non-traumatic but challenging life changes such as the death of a loved one, separation or work-related problems (e.g. bullying, work overload, job loss; Karatzias et al., 2021). Although such events are not unusual in the course of life, they may still lead to clinically significant psychological impairment when coping resources are insufficient.

Both major diagnostic classification systems—the International Classification of Diseases (ICD; e.g. World Health Organisation, 2018) and the Diagnostic and Statistical Manual of Mental Disorders (DSM; e.g. APA, 2013)—include AjD as a distinct mental health diagnosis. Although the diagnostic criteria differ between taxonomies and have been revised over time, they share a common assumption: stressful events can lead to clinically significant psychological distress and functional impairment. AjD differs from other mental health disorders less by its specific symptoms (e.g. distressing thoughts) than by the explicit attribution of symptoms to an identifiable psychosocial stressor. In earlier diagnostic systems, however, AjD was characterised by the absence of disorder-specific symptom criteria, which complicated its differentiation from subsyndromal depressive and anxiety disorders as well as from non-pathological stress reactions. The AjD concept has therefore been criticised for its conceptual vagueness, limited reliability, and restricted discriminant validity (e.g. Baumeister et al., 2009; Reed et al., 2011).

In response to such criticisms, the ICD-11 introduced a revised definition of AjD that provides a more clearly articulated symptom-based framework by specifying two core symptom clusters: preoccupation with the stressor (i.e. excessive mental preoccupation with the stressor or its consequences) and failure to adapt (e.g. persistent difficulties in recovery, sleep disturbances or concentration problems). Additional accessory symptoms may include avoidance, depressive and anxious symptoms, and impulse control difficulties (World Health Organisation, 2018). Symptoms must not be better explained by another mental disorder (e.g. a mood disorder, another disorder specifically associated with stress).

With these changes, the ICD-11 revision has reignited scientific interest in the diagnosis of AjD (Bachem & Casey, 2018). For the first time, disorder-specific assessment tools were developed and validated to measure AjD symptom severity, including the self-report questionnaire Adjustment Disorder - New Module 20 (ADNM-20; Glaesmer et al., 2015) and the structured Composite International Diagnostic Interview - Module Adjustment Disorder (CIDI-AD; Perkonigg et al., 2021). In both instruments, respondents are first asked to select the event that causes them the greatest distress from a list of non-traumatic psychosocial stressors, followed by an assessment of associated symptom severity. Accordingly, the operationalisation of AjD as a stress-related disorder is based on the assumption of a link between a specific triggering event and the reported symptoms, and consequently assumes that individuals can identify and report the primary trigger for their symptoms.

Importantly, recent studies on the ICD-11 AjD concept indicate that exposure to stressful life events is highly prevalent and that multiple concurrent stressors are common rather than exceptional, both in community samples and in clinical settings (e.g. Perkonigg et al., 2018; Zelviene et al., 2017; Bachem & Casey, 2018). In psychosomatic rehabilitation in particular, patients often face accumulated constellations of stressors, such as work-related strain, family conflict, and health problems (Zwerenz et al., 2004; Jerg-Bretzke et al., 2020). This high stressor density may complicate the identification of a single ‘main’ event and raises the question of how the ICD-11 requirement of a clearly attributable stressor operates in such contexts.

Against this background, examining how psychosocial stressors are identified across different assessment approaches is particularly relevant in psychosomatic rehabilitation settings. Both the ADNM-20 and the CIDI-AD are grounded in the same ICD-11 definition of AjD but differ in their assessment modality, with the former relying on self-report and the latter on a structured clinical interview. Comparing stressor identification across these approaches allows an examination of how stressful life events are reported across methods when multiple concurrent stressors are present. Such comparisons may provide insight into the practical implementation of the ICD-11 stressor-based concept of AjD-related symptomatology in complex clinical contexts, without presupposing consistency or inconsistency across methods.

The present study focuses on the two first disorder-specific ICD-11 based measures of AjD symptom patterns, the ADNM-20 and the CIDI-AD. Specifically, it aims to (1) identify the types of life events most frequently reported as triggers of AjD symptoms in psychosomatic rehabilitation and (2) examine the degree of correspondence between stressor categories identified across self-report- and interview-based assessment methods.

## Methods

### Procedure and sample

The present analyses are based on data from the study *Adjustment Disorders in the Context of Psychosomatic Rehabilitation* (Kupferschmitt, 2024). This multicenter, non-randomised, controlled field study employed a longitudinal design and included standardised assessment of stressors associated with AjD symptoms and symptom patterns using both self-report questionnaires (ADNM-20) and structured interviews (CIDI-AD) at the beginning and end of rehabilitation. The target sample size was n ≥ 2000 for the questionnaire assessment and n ≥ 300 for the clinical interviews (plus an expected 20% dropout, n = 60). Data collection took place between January 2019 and March 2021 in three German rehabilitation clinics. Approval for the research was granted by the Ethics Committee of the Brandenburg State Medical Association (reference AS 158 (bB)/2019) in December 2019.

All clinics implemented multimodal rehabilitation concepts including individual and group cognitive-behavioural psychotherapy (CBT), individualised endurance and exercise therapy (shown to have antidepressant effects; Puetz et al., 2008), relaxation techniques, occupational or art therapy, medical examinations, physiotherapy and social-medical counselling. The integration of multiple interventions, embedded within a group therapy context has been shown to promote behavioural activation (Melicherova et al., 2024) and overall well-being (Fava et al., 2017).

Between January 2019 and March 2021, N = 2063 consecutively admitted patients were included in the questionnaire sample. The ADNM-20 was administered at admission and discharge, while the CIDI-AD interview was conducted after obtaining written informed consent, typically on the same day as the AjD self-report questionnaire (ADNM-20) or the following day. The interview was conducted blindly by experienced clinicians trained in structured interviewing. In total, 388 interviews were scheduled, with 14 participants discontinued, and one declined participation. None of the patients refused to complete the questionnaire assessment. Consequently, 373 interviews were included for data analysis. Clinical diagnoses were based on ICD-10 as it is currently used in the billing of health insurance services in Germany. The ICD-10 diagnoses were made within the first two weeks of admission by the treating psychological psychotherapist or medical psychotherapist according to AMDP criteria, always reviewed by an experienced specialist (4-eyes principle). The diagnostic process was carried out by expert judgement based on psychopathological expertise, using observational information (e.g. formal thought processing, modulation of mood) and the the overal symptom pattern.

The research was approved by the Ethics Committee of the Brandenburg State Medical Association (Reference AS 158 (bB)/2019) in December 2019.

### Measures

**Adjustment disorder self-report questionnaire (ADNM-20).** The Adjustment Disorder—New Module 20 (ADNM-20) questionnaire (Einsle et al., 2010; Glaesmer et al., 2015) is a self-report instrument designed to assess the two core symptoms of ICD-11 AjD (preoccupation and failure to adapt) as well as accessory symptoms stated in ICD-11. The questionnaire consists of two parts: The first part consists of a list of 17 common and widespread psychosocial stressors (e.g. job loss, family stressors such as conflicts or separations, own illness or illness of a close person, various other stressors such as moving house), which occurred during the last two years. Two additional items were added to account for private and occupational stressors related to the COVID-19 pandemic.

The second part assesses whether and to what extent AjD-relevant symptoms are present (e.g. I cannot stop thinking about the stressful event(s)). The responses are given on a 4-point Likert scale ranging from 1 never to 4 often (scale range 20−80). The 20 items form six subscales that correspond to the two core symptom clusters of preoccupation (4 items) and failure to adapt (4 items), as well as the four additional domains: avoidance (4 items), depressive mood (3 items), anxiety (2 items), and impulse-control difficulties (3 items). Subscale scores (sum or mean values) represent the severity of the symptoms, with higher scores reflecting higher distress. The total score can be used as a global indicator of AjD-relevant symptom severity, supported by factor-analytic findings that point towards a uni-faceted concept, in which the individual factors are highly correlated (Glaesmer et al., 2015; Lorenz et al., 2018). In the present study, Cronbach’s alpha was .93 for preoccupation, *α* = .86 for failure to adapt and *α* = .96 for the ADNM-20 total scale.

The ADNM-20 allows two complementary evaluation approaches. First, AjD symptom severity can be quantified using the total score, with a cut-off of ≥45.7 indicating at least moderate and clinically relevant symptom severity. Second, a categorical diagnostic classification consistent with ICD-11 criteria can be derived by applying the ADNM-20 diagnostic algorithm. This algorithm requires fulfilment of the event criterion as well as the presence of both core symptom clusters: preoccupation (at least one item ≥3 and two items ≥2 out of five items) and failure to adapt (at least one item ≥3 and two items ≥2 out of four items) must be present (Lorenz et al., 2016).

**Adjustment disorder clinical interview (CIDI-AD).** Structured clinical interviews are considered the gold standard for diagnosing mental disorders, with the World Mental Health Composite International Diagnostic Interview (WHO WMH-CIDI) being one of the most widely used and validated instrument (Hoyer et al., 2020; Kessler & Üstün, 2004; Perkonigg et al., 2021; Reed et al., 1998; Wittchen, 1994). The CIDI-AD is an add-on module to the standardised WHO WMH-CIDI (CIDI; Perkonigg et al., 2018b), developed specifically to assess ICD-11 AjD symptom patterns. This extension was necessary because the ICD-11 introduced disorder-specific diagnostic criteria for AjD, including the two core symptom clusters of preoccupation with the stressor and failure to adapt, which had not been operationalised in previous CIDI versions.

In terms of content and structure, the CIDI-AD parallels the ADNM-20. It begins with a stressor checklist covering the same domains as the ADNM-20 (e.g. work, family, health, financial, and housing problems), supplemented by the COVID-19-related items. First, the event list was read out event by event, and respondents were asked to indicate which of the events they had experienced. Respondents were then asked to select the worst of the events they had experienced. The worst event was then specified in terms of onset, frequency and duration, and was used as a reference for the subsequent symptom assessment.

Moreover, the same AjD symptoms correspond to the subscales of preoccupation (5 items), failure to adapt (6 items), avoidance (3 items), depressive moods (4 items), anxiety (3 items) and impulse control disorder (5 items) are then assessed by the interviewer. Responses are coded dichotomously (yes/no). Additional items assess time of symptom onset (within 1 month after the stressor), symptom frequency and intensity, and functional impairment in work, social, and leisure contexts. The diagnostic algorithm requires the presence of a stressor, at least one *preoccupation* symptom and two *failure to adapt* symptoms, onset within one month, and significant functional impairment.

The CIDI-AD has demonstrated good interrater reliability for the ICD-11 AjD diagnosis (*κ* = .81), acceptable internal consistency (*α* = .43–.80 across symptom domains), and construct validity supported by latent class and confirmatory factor analyses (Perkonigg et al., 2021). In the present study, Cronbach’s alpha was .78 for preoccupation, *α* = .66 for failure to adapt and *α* = .81 for the CIDI-AD total symptoms scale.

### Statistical analyses

All statistical analyses were performed using SPSS Statistics 28 for Windows. Descriptive statistics (frequency analyses) were calculated to describe the sample characteristics, determine the frequency of AjD diagnoses according to the self-report- (ADNM-20) and interview-based assessment methods (CIDI-AD), as well as to examine the triggering events (event lists) in more detail. Because the event lists of the ADNM-20 and the CIDI-AD are not completely identical, even though they cover comparable life domains, individual stressors were grouped into conceptually equivalent categories to allow comparison and to enable consistency of stressor reporting across instruments. The events were assigned to categories as follows: work-related stressors (e.g. job loss, time pressure), own illness, family-related stressors (e.g. family conflict, separation/divorcee), illness/death in the family, and ‘other stressors’, including events that could not be assigned elsewhere (e.g. giving up an important leisure activity, moving house). The assignment of individual events to these event categories is presented in Table 2. The consistency of responses was calculated as the percentage of individuals who consistently reported an event from the same category in both the ADNM-20 and CIDI-AD.

## Ethics statement

This research was approved by the Ethics Committee of the Brandenburg Medical Association (identification AS 158(bB)/2019) in December 2019.

Prior to study participation patients were informed about the study objectives and the study procedure in a personal information consultation and had received standardised participant information sheets. Then, voluntary written informed consent for study participation and storage, evaluation and transfer of study-related data was obtained from each study participant by research associates of the respective study centre. Withdrawal of written consent was possible at any time, without giving reasons.

## Results

### Sample description

The total sample comprised N = 2063 patients undergoing psychosomatic rehabilitation. A consecutive subsample of *n* = 373 (18.08%) also completed a clinical interview. In the interview subsample, the mean age was 54.75 years (SD 7.37), and 37.8% of participants were men while 62.2% were women. The mean duration of inpatient rehabilitation was 39.3 days (SD 8.7). A detailed description of the sample is provided in Table 1.

**Table 1.** Descriptive characteristics of the total sample and the interview subsample.

	Total Sample (*N* = 2063)	Interview Subsample (*n* = 373)	*p*
*Demographic variables*			
Age, years M (SD)	52.72 (8.96)	54.75 (7.37)	.175
Gender (% female)	64.9	62.2	.273
Married (%)	50.8	48.8	.399
Compulsory schooling/secondary school without vocational training (%)	3.6	3.5	.088
Completed vocational training/technical college (%)	60.3	61.4	.137
High school diploma/technical college (%)	25.5	23.9	.408
University/higher education degree (%)	9.9	11.0	.308
*Most common* p*rimary referral mental health diagnoses (according to ICD-10)*			
Depressive disorder (%)	48.5	46.7	.313
Anxiety disorder (%)	17.5	17.8	.356
Adjustment disorder (%)	15.7	17.2	.375
Somatoform disorder (%)	6.4	6.6	.495
PTSD (%)	3.9	5.2	.493
other (F0, F17, F44, F51, F54, F90, F98) (%)	5.7	5.4	.387
personality disorder (%)	2.3	1.1	.333
*Job-related characteristics*			
Employment relationship exists (%)	70.6	63.3	.128
Ability to work (% fit for work)	37.1	45.3	.137
*Duration of sick leave within last 12 months*			
none	6.7	6.3	.457
<3 months (%)	30.2	28.6	.312
3–6 months (%)	11.5	12.7	.309
>6 months (%)	48.2	48.8	.416
Unfit for work (%)	3.4	3.6	.408

Note: T-test für Age, χ^2^ for the remaining variables.

Using the ADNM-20 total score criterion, 75.9% of the overall sample showed clinically relevant symptom severity, while in the interview subsample 86.1% reached this severity threshold. When applying the ICD-11–based ADNM-20 diagnostic algorithm, 66.9% of the total sample met criteria for probable AjD according to ICD-11-based instruments. Based on the CIDI-AD interview, 51.5% of patients in the interview subsample met criteria for AjD according to ICD-11-based instruments. The comparatively high proportion of participants fulfilling ICD-11 AjD criteria in the CIDI-AD should be interpreted in light of the study context. The interview algorithm focused specifically on AjD symptom criteria and stressor exposure but did not apply a full diagnostic hierarchy assessment across all competing mental disorders at the time of interview. Consequently, interview-based AjD classifications represent probable ICD-11 AjD symptom constellations rather than final differential clinical diagnoses.

### Frequency and distribution of stressful life events in the ADNM-20 and CIDI-AD

Across both assessment instruments, patients reported a high overall burden of stressful life events, with stressors occurring in multiple life domains (see Table 2). In both the ADNM-20 questionnaire and the CIDI-AD interview, work-related stressors emerged as the most frequently reported category and were most often identified as the most stressful events.

**Table 2.** Descriptive statistics of stressor categories assessed by the ADNM-20 and CIDI-AD.

		ADNM-20 (*n* = 373)		CIDI-AD (*n* = 373)	
Category	Stressor	Experienced (%)	Reported as most stressful event (%)	Experienced (%)	Reported as most stressful event (%)
Work-	Overall	98	35	78	39
	Job loss	24	2	14	7
	Workplace conflicts	83	17	53	16
	Workload	84	7	59	14
	Deadline/time pressure	91	7	−	−
	Retirement	10	1	5	0
	COVID-19-related occupational difficulties	31	1	28	2
Family	Overall	83	15	66	13
	Family conflicts	67	9	41	8
	Separation/divorcee	27	5	9	3
	COVID-19-related family difficulties	51	1	40	2
Own illness	Serious illness of oneself	77	23	57	22
Death/illness (family member)	Total	77	20	57	17
	Illness of a family member	60	8	45	8
	Death of a close relative	57	12	34	9
Other stressor	Overall	88	7	74	10
	Conflicts with neighbours	26	1	12	0
	Conflicts with authorities	−	−	21	2
	Moving house	17	0	9	0
	Financial problems	51	1	19	1
	Accident	9	0	5	0
	Assault	6	1	2	0
	Giving up an important leisure activity	48	1	45	2
	Other	57	3	24	5
Total number of events		3.242		1.852	

In the ADNM-20, nearly all patients reported at least one work-related stressor, and more than one third identified such an event as the most stressful. In contrast, work-related stressors were reported less frequently in the CIDI-AD interview; however, they remained the most prevalent stressor category and were most commonly rated as the most stressful.

Family-related stressors represented the second most frequent stressor domain in both instruments. Although commonly reported, family stressors were less often identified as the most stressful events compared to work-related stressors. The overall frequency of family stressors was higher in the ADNM-20 than in the CIDI-AD, whereas the proportion of family events rated as most stressful was comparable across instruments.

Health-related stressors, including serious illness of the patient or illness or death of close relatives, were reported by a substantial proportion of participants in both assessments. Although less prevalent than work- and family-related stressors, these events were relatively often identified as the most stressful events.

Finally, stressors from the ‘Other’ category were frequently reported in both instruments but rarely selected as the most stressful. Overall, higher frequencies across stressor domains were observed in the ADNM-20, whereas the CIDI-AD showed lower frequencies, particularly for work- and family-related stressors.

### Agreement and consistency of stressor reports

**Figure 1.**
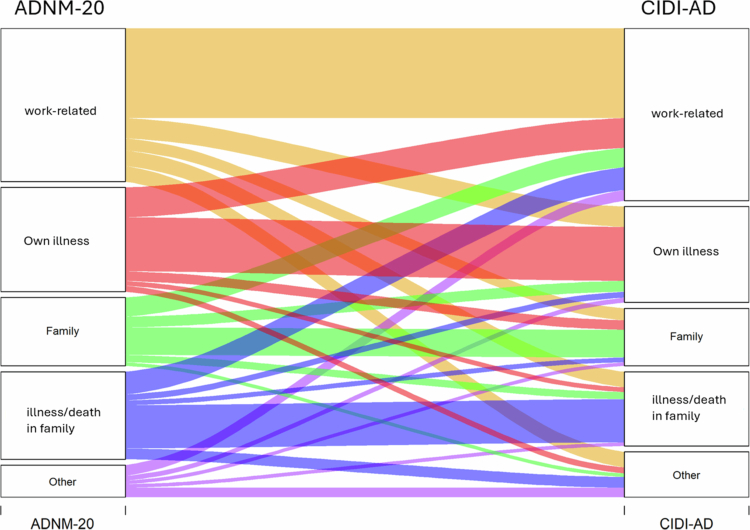
Alluvial Plot of the Most Stressful Events Reported in the ADNM-20 and CIDI-AD. Note: Colours correspond to stressor categories (orange = work-related, red = own illness, green = family, blue = illness/death in family, purple = other). Band widths are proportional to the number of participants.

On average, participants reported nine stressful events in the ADNM-20 stressor list (range 1–19) and five stressful events in the CIDI-AD event list (range 1–19). Among the 373 individuals who completed both the questionnaire and the interview, only 49% reported the same primary stressor across instruments (see Table 3a and Figure 1). For example, in the ADNM-20, 35% reported a work-related stressor as their most stressful event, compared to 39% in the CIDI-AD interview. However, agreement at the individual level was limited: among the 227 participants who reported a work-related stressor in the ADNM-20, only 52% did so in the CIDI-AD. The remaining participants selected a different primary stressor in the interview, including own illness, illness or death of a family member, family-related stress, or stressors classified as ‘other’.

**Table 3a.** Most stressful events according to patient reports in the ADNM-20 and CIDI-AD assesments.

	CIDI (%)				
ADNM (%)	Family	Work	Own illness	Death/illness family member	Other stressor
Family	**50.0**	8.7	11.3	9.7	10.4
Work	14.3	**50.7**	26.3	21.0	41.7
Own illness	19.0	18.1	**53.8**	8.1	16.7
Death/illness Family member	11.9	13.8	6.3	**56.5**	16.7
Other stressor	4.8	7.2	2.5	4.8	**10.4**

Significance was for all <.05.

As a sensitivity analysis, the main comparisons were repeated in the subgroup of patients with a clinically diagnosed AjD after exclusion of comorbid diagnoses (20.84% of the total sample). Although statistical power was reduced due to the reduced sample size, the overall pattern of multiple concurrent stressors and limited agreement between ADNM-20 and CIDI-AD remained comparable to that of the full sample (see Table 3b).

**Table 3b.** Most stressful events in patients with clinical diagnosis of adjustment disorder (*n* = 60).

CIDI (%)					
ADNM (%)	Family	Work	Own illness	Death/illness family member	Other stressor
Family	**50.0**	4.0	9.1	0.0	11.1
Work	0.0	**64.0**	45.5	0.0	44.4
Own illness	16.7	0.0	**45.5**	22.2	0.0
Death/illness Family member	16.7	20.0	0.0	**77.8**	0.0
Other stressor	16.7	8.0	0.0	0.0	**33.3**

Significance was for all <.05.

## Discussion

In this study, we examined the distribution of stressor domains linked to ICD-11 AjD symptoms and the consistency with which patients prioritised a primary stressor when stressor information was elicited using two different assessment approaches (ADNM-20 checklist; CIDI-AD interview) in a psychosomatic rehabilitation sample. Importantly, the present study does not assume that all observed stress-related symptoms reflect categorically defined adjustment disorder. Rather, the ICD-11 AjD framework was used as a structured model to examine stressor-related symptom processes in a diagnostically heterogeneous rehabilitation population. Work-related, health-related, and family stressors emerged as the most frequent triggering events of AjD symptoms, with work stressors predominating. However, only about half of the participants identified the same primary across formats, suggesting that identifying and prioritising of a single ‘main’ stressor may be challenging in complex clinical contexts characterised by multiple concurrent stressors.

In this rehabilitation sample, symptoms characteristic of ICD-11 AjD were highly prevalent, underscoring the clinical relevance of AjD-related symptomatology in this patient group, where stress-related and affective conditions frequently cooccur. It is important to note that many participants met criteria for other mental disorders. Under ICD-11, AjD is typically diagnosed only when symptoms are not better accounted for by another condition, such as a mood or stress-related disorder. The present study, however, did not seek to establish formal diagnoses but to examine the nature and consistency of stressors typically associated with AjD symptomatology in a complex clinical population. This perspective is consistent with prior AjD research in clinical populations that has examined stress-related processes beyond strict categorical diagnoses (e.g. Shevlin et al., 2020; Bachem et al., 2019).

### Triggering events in the rehabilitation context

Work-related stressors emerged as the most frequently reported triggers of AjD symptoms across both assessment methods. This finding aligns with prior research indicating that occupational problems, alongside family conflicts and health-related stressors, represent central sources of psychological burden (Perkonigg et al., 2018a). In psychosomatic rehabilitation, the prominence of work-related stressors is unsurprising, as patients often report elevated occupational strain, workplace conflicts, and negative perceptions of work demands compared to the general population (Zwerenz et al., 2004). From a clinical-contextual perspective, stressors such as job insecurity, performance pressure or structural workplace conflicts may reflect prolonged occupational strain and concerns about professional stability at the time of admission. Accordingly, the high prevalence of work-related triggers underscores the importance of assessing and addressing occupational stressors in rehabilitation settings, for example through work-oriented psychotherapeutic approaches or social-medical counselling (e.g. Arends et al., 2012; Baumeister et al., 2022). The predominance of occupational stressors also raises questions regarding conceptual overlap with burnout-related processes. Although burnout is not classified as a mental disorder in ICD-11, chronic occupational stress, exhaustion, and impaired adaptation may partially overlap phenomenologically with AjD symptom presentations in rehabilitation populations. Future studies should therefore further investigate the boundaries and intersections between work-related AjD symptomatology and burnout constructs.

Beyond occupational demands, patients also frequently reported relationship breakups, persistent family conflicts, and severe somatic illness, all of which are recognised as prototypical stressors associated with AjD symptoms. In line with this broader stressor spectrum, rehabilitation settings should not only focus on occupational strain but also systematically assess and address non-work stressors, for instance by integrating modules on coping with illness, relational losses, and migration- or financially related adversity into psychotherapeutic and social-medical interventions (Lorenz et al., 2018; Maercker & Eberle, 2022; O’Donnell et al., 2019; Yue & Lau, 2025).

### Consistency of triggering events

On average, participants listed a greater number of stressors in the ADNM-20 than in the CIDI-AD, indicating a higher event load in the questionnaire format. Because both instruments were administered within the same clinical setting and time frame, this difference is unlikely to reflect contextual factors. Instead, it may be attributable to method-related differences in information elicitation. Written checklists may facilitate broader recall and endorsement of multiple, whereas structured interviews, through contextual probing, may narrow attention to the most salient events (Duggal et al., 2000; Gorman, 1993; Lewinsohn et al., 2003).

Beyond the number of reported stressors, agreement regarding the primary trigger was limited: only about half of participants identified the same event as their primary trigger across assessment approaches. This finding highlights the practical difficulty of prioritising a single ‘most stressful’ event in clinical contexts characterised by multiple co-occurring stressors. Rather than indicating reporting unreliability, the pattern may reflect the fact that stressors are often experienced as overlapping and mutually reenforcing, making it difficult to isolate one event as uniquely causal. During the assessment process, some participants spontaneously expressed difficulty identifying a single ‘most stressful’ event, further illustrating the challenge of prioritising one stressor in the presence of multiple concurrent burdens.

These findings raise broader conceptual questions regarding the assumption that AjD-relevant symptoms can be clearly linked to a single index event. In complex clinical settings, maladaptive stress responses may instead arise within constellations of ongoing, interacting stressors. At the same time, differences in assessment format likely influence how prioritisation is expressed, underscoring the need to disentangle conceptual from method-related effects in future research.

Another possible explanation for differences between questionnaire- and interview-based stressor prioritisation may involve assessment-order effects. Because participants completed the ADNM-20 before the CIDI-AD interview, prior reflection on stressful events may have influenced subsequent stressor selection during the interview process. At the same time, the interview format may also have encouraged re-evaluation and contextual elaboration of stressor salience. Future studies could experimentally vary assessment order or include cognitive interviewing approaches to better understand how individuals prioritise stressors across assessment contexts.

Importantly, the present sample included many individuals who met criteria for other mental disorders and would not formally receive an AjD diagnosis under ICD-11. However, exposure to multiple concurrent stressors is not unique to diagnostically mixed rehabilitation samples. Population-based studies have shown that individuals often report several critical life events within a limited timeframe (e.g. Zelviene et al., 2020), and clinical studies of confirmed AjD cases report exposure to multiple stressors (e.g. Eimontas et al., 2018; Vancappel et al., 2024). Importantly, the same pattern was observed in sensitivity analyses restricted to patients with a primary clinical diagnosis of AjD. Together, these findings suggest that stressor overlap may represent a general feature of maladaptive stress responses rather than a context-specific artefact.

### Limitations

Several limitations of the study should be noted. First, the sample consisted of patients undergoing psychosomatic rehabilitation, which may limit generalisability to outpatient or community populations. Second, although AjD-related symptoms were assessed according to ICD-11 criteria, the formal exclusion hierarchy was not applied, and many participants met criteria for additional mental disorders. Consequently, the findings pertain to AjD-related stressor patterns in a diagnostically complex clinical population rather than to categorically defined ‘pure’ AjD cases. Third, stressors were assessed retrospectively via questionnaire and interview, which may be subject to recall bias and influenced by current symptom states, potentially affecting both the number and prioritisation of reported stressors. Future studies should therefore examine stressor constellations and their stability over time and across clinical contexts in diagnostically confirmed and population-based samples to determine the generalisability of these findings.

## Conclusion

The present findings suggest that in psychosomatic rehabilitation settings characterised by multiple concurrent stressors, AjD-related symptomatology may be embedded in broader constellations of stress exposure rather than being linked to a single discrete event. The limited consistency in stressor prioritisation observed in this study indicates that the practical application of the ICD-11 stressor requirement may be more complex in real-world clinical contexts than implied by a one-to-one stressor–response model. Importantly, these observations do not challenge the core symptom-based conceptualisation of AjD within ICD-11. Rather, they suggest that the operationalisation of the stressor criterion may become increasingly complex in settings characterised by cumulative and overlapping stress exposure.

## Data Availability

Since the data are data on insured persons of the German Pension Insurance and strict data protection exists, these data are not publicly accessible, but can be made available by the corresponding author in a form adjusted for personal data upon justified request. https://doi.org/10.57760/sciencedb.27740.
